# Sequential screening for lung cancer in a high-risk group: randomised controlled trial: LungSEARCH: a randomised controlled trial of Surveillance using sputum and imaging for the EARly detection of lung Cancer in a High-risk group

**DOI:** 10.1183/13993003.00581-2019

**Published:** 2019-10-17

**Authors:** Stephen G. Spiro, Pallav L. Shah, Robert C. Rintoul, Jeremy George, Samuel Janes, Matthew Callister, Marco Novelli, Penny Shaw, Gabrijela Kocjan, Chris Griffiths, Mary Falzon, Richard Booton, Nicholas Magee, Michael Peake, Paul Dhillon, Kishore Sridharan, Andrew G. Nicholson, Simon Padley, Magali N. Taylor, Asia Ahmed, Jack Allen, Yenting Ngai, Nyasha Chinyanganya, Victoria Ashford-Turner, Sarah Lewis, Dahmane Oukrif, Pamela Rabbitts, Nicholas Counsell, Allan Hackshaw

**Affiliations:** 1 Dept of Respiratory Medicine, University College Hospital, London, UK; 2 Dept of Respiratory Medicine, Royal Brompton Hospital, Chelsea and Westminster Hospital and Imperial College London, London, UK; 3 Dept of Oncology, Royal Papworth Hospital and University of Cambridge, Cambridge, UK; 4 UCL Respiratory, Dept of Medicine, University College London, London, UK; 5 Dept of Respiratory Medicine, Leeds Teaching Hospitals NHS Trust, Leeds, UK; 6 Cellular Pathology, University College Hospital, London, UK; 7 Radiology (Imaging), University College Hospital, London, UK; 8 Institute of Population Health Sciences, Barts and the London School of Medicine and Dentistry, Queen Mary University of London, London, UK; 9 Lung Cancer and Thoracic Surgery Directorate, Manchester University NHS Trust and University of Manchester, Manchester, UK; 10 Respiratory Medicine, Belfast City Hospital, Belfast, UK; 11 Dept of Immunity, Infection and Inflammation, University of Leicester, Leicester, UK; 12 Centre for Cancer Outcomes, University College London Hospitals NHS Foundation Trust, London, UK; 13 Respiratory Medicine, University Hospitals Coventry and Warwickshire, Coventry, UK; 14 Dept of Thoracic Medicine, Sunderland Royal Hospital, Sunderland, UK; 15 Dept of Histopathology, Royal Brompton Hospital and Harefield NHS Foundation Trust and National Heart and Lung Institute, London, UK; 16 Radiology, Royal Brompton Hospital and National Heart and Lung Institute, Imperial College London, London, UK; 17 Cancer Research UK and UCL Cancer Trials Centre, London, UK; 18 Cardio-Respiratory Medicine, Leeds Teaching Hospitals NHS Trust, Leeds, UK; 19 Research and Development, Royal Papworth Hospital, Cambridge, UK; 20 Dept of Pathology, University College Hospital, London, UK; 21 Leeds Institute of Cancer and Pathology (LICAP), University of Leeds, Leeds, UK; 22 These authors are joint lead authors

## Abstract

**Background:**

Low-dose computed tomography (LDCT) screening detects early-stage lung cancer and reduces mortality. We proposed a sequential approach targeted to a high-risk group as a potentially efficient screening strategy.

**Methods:**

LungSEARCH was a national multicentre randomised trial. Current/ex-smokers with mild/moderate chronic obstructive pulmonary disease (COPD) were allocated (1:1) to have 5 years surveillance or not. Screened participants provided annual sputum samples for cytology and cytometry, and if abnormal were offered annual LDCT and autofluorescence bronchoscopy (AFB). Those with normal sputum provided annual samples. The primary end-point was the percentage of lung cancers diagnosed at stage I/II (nonsmall cell) or limited disease (small cell).

**Results:**

1568 participants were randomised during 2007–2011 from 10 UK centres. 85.2% of those screened provided an adequate baseline sputum sample. There were 42 lung cancers among 785 screened individuals and 36 lung cancers among 783 controls. 54.8% (23 out of 42) of screened individuals *versus* 45.2% (14 out of 31) of controls with known staging were diagnosed with early-stage disease (one-sided p=0.24). Relative risk was 1.21 (95% CI 0.75–1.95) or 0.82 (95% CI 0.52–1.31) for early-stage or advanced cancers, respectively. Overall sensitivity for sputum (in those randomised to surveillance) was low (40.5%) with a cumulative false-positive rate (FPR) of 32.8%. 55% of cancers had normal sputum results throughout. Among sputum-positive individuals who had AFB, sensitivity was 45.5% and cumulative FPR was 39.5%; the corresponding measures for those who had LDCT were 100% and 16.1%, respectively.

**Conclusions:**

Our sequential strategy, using sputum cytology/cytometry to select high-risk individuals for AFB and LDCT, did not lead to a clear stage shift and did not improve the efficiency of lung cancer screening.

## Abstract 2

**While low-dose CT is now preferred for lung cancer screening, our randomised trial of smokers with COPD showed that a proposed sequential policy using sputum testing to select who receives low-dose CT and autofluorescence bronchoscopy was ineffective** http://bit.ly/2JZujnx

## Introduction

Lung cancer is associated with poor survival because most cases are diagnosed at a late stage. However, early detection with intended curative treatments can have an 80% 1-year survival rate for stage I disease [1].

During the 2000s, several randomised trials were developed to evaluate low-dose computed tomography (LDCT) [2]. Expected major issues with LDCT screening included affordability and high false-positive rates (FPRs) (which can be reduced through improved management of pulmonary nodules) [3]. Furthermore, LDCT might miss early squamous cell tumours located in the central airways [4].

Two major LDCT trials (the US National Lung Screening Trial (NLST) and the NELSON study) now show a clear reduction in lung cancer mortality among current/ex-smokers who had annual LDCT compared with either chest radiography or no screening [5, 6]. LDCT screening is recommended in the USA [7] and suggested for Europe [8]. However, uptake in the USA is low (<5% of those eligible) [9, 10]. Our LungSEARCH study was developed in 2006, long before NLST and NELSON were published [5, 6]. We proposed a different strategy to make screening more efficient. Instead of offering a single screening test, we created a novel approach of sequential screening (using sputum and imaging) and in a particularly high-risk group, *i.e.* current/ex-smokers with chronic obstructive pulmonary disease (COPD), based on promising evidence for the component tests.

COPD is correlated with lung cancer risk, and is an independent risk factor to smoking and other characteristics [11, 12]. Decreasing lung function (using Global Initiative for Chronic Obstructive Lung Disease (GOLD) criteria) is associated with increasingly worse survival [13, 14]. Therefore, targeted lung cancer screening among individuals with COPD is appealing [11, 15–17].

Sputum cytology is a noninvasive and nonradiological test for lung disease, especially central airway tumours. Sample procurement can be done at home without specialist equipment. Many smokers (particularly those with COPD) produce more sputum, containing exfoliated cells from the bronchial tree. There is an established association between having abnormal sputum cytology and lung cancer [18, 19], although the earlier randomised trials of cytology failed to reduce lung cancer mortality [20]. However, modern cytology methods have better sensitivity. Another sputum test involves computer-assisted image analysis (automated image cytometry), which quantitatively analyses the nuclear structure and DNA content of individual cells, distinguishing normal from suspicious cells [21–23]. In a large study of smokers, 80% of lung cancers with sputum samples had abnormal cytometry compared with only 4% who had abnormal cytology [21]. We hypothesised that the high-performance sensitivities expected using modern cytology/cytometry would miss few cancers as a first screening test.

Autofluorescence bronchoscopy (AFB) is an optical imaging technique that compares fluorescence properties between normal and malignant/pre-malignant bronchial mucosa [24–26]. AFB has a sensitivity for early-stage lung cancer of 44–82% compared with 9–58% using conventional white light bronchoscopy [26]. The sensitivity for detecting abnormal lesions using AFB with white light could be two times that using white light alone [27]. In a prior study of individuals with pre-invasive lesions, 73% had one or more high-grade lesions and one in six of these lesions progressed to invasive carcinoma [28, 29].

LungSEARCH evaluated sequential testing for detecting lung cancer in a high-risk group, in which a cheap first screen is used to select who is offered LDCT and AFB. To date, it is the only randomised lung cancer screening study to triage participants.

## Methods

### Design and participants

LungSEARCH was a national multicentre randomised trial. The objective was to examine whether annual surveillance of individuals at high risk of lung cancer (current/ex-smokers with COPD) can lead to a shift in cancer stage at diagnosis.

Participants were identified primarily from general practice. A research nurse visited each practice to perform an electronic search of their COPD register and those potentially eligible were invited by telephone to attend for baseline assessments. We also approached participants within outpatient COPD or pulmonary rehabilitation hospital clinics in which the trial investigators worked.

Baseline COPD (by spirometry) was classified according to GOLD criteria as mild (forced expiratory volume in 1 s (FEV_1_)/forced vital capacity (FVC) <70%; FEV_1_ ≥80% predicted) or moderate (FEV_1_/FVC <70%; FEV_1_ 50–80% predicted) [30, 31]. Those with mild/moderate COPD were eligible for the trial if they currently smoked or were ex-smokers who had quit within 8 years (agreed by the investigators to still have a high risk of lung cancer), and both groups had ≥20 pack-years and/or had smoked for ≥20 years (thresholds often used in studies at the time), had no history of malignant disease during the previous 5 years, and were without serious comorbidities. The trial had multicentre ethics approval and participants gave written informed consent. The trial is registered at the ISRCTN registry with identifier ISRCTN80745975.

### Randomisation

Participants were randomised (1:1) to have annual screening/surveillance or not (controls). Research nurses telephoned the Cancer Trials Centre (London, UK), where the random allocation (minimisation) was performed by computer, stratified by location, 10-year age bands, sex, smoking status (ex-smoker or current smoker) and mild/moderate COPD.

### Procedures

Individuals in the control arm had no trial-specific procedures, but to encourage study continuation they were offered an exit chest radiograph 5 years post-randomisation (or sooner if they withdrew earlier) if they had not developed lung cancer. This was also offered to the screened group.

Individuals in the screened group had sputum cytology and cytometry as initial tests, and only those with abnormal findings were offered LDCT and AFB, expecting that these in combination would be better than either alone at finding cancer in the central airways (by AFB) and peripheral airways (by LDCT) (supplementary figure S1). The three component tests are described in the supplementary material. Screened individuals posted sputum samples to the central laboratory for assessment, annually. Those with normal cytology/cytometry provided sputum samples the following year. Unless participants formally withdrew from the trial, they were asked to provide sputum annually even if they had not done so previously.

Specimens obtained *via* AFB were categorised as positive/abnormal if the cells exhibited squamous metaplasia, mild to severe dysplasia, carcinoma *in situ* or carcinoma. LDCT (target radiation dose <2 mSv) was conducted without contrast. A positive/abnormal LDCT (nodule size ≥9 mm) could initiate cancer investigations according to local practice. Individuals with both normal AFB and LDCT continued to have these tests annually. Individuals with abnormal AFB or LDCT, not indicative of invasive cancer, could be seen 4–6 months later, depending on nodule size. Neither group provided further sputum samples.

All participants were flagged with established cancer registries (Health and Social Care Information Centre in England or the Northern Ireland Cancer Registry); notifications were received until April 2018. Research nurses also periodically checked patient records for cancer diagnoses. These two sources provided the cancer notifications; stage and histology at diagnosis were then manually retrieved from medical records.

### Outcomes

The primary outcome was the proportion of lung cancers diagnosed at an early stage, an end-point used previously [32, 33]: stage I/II for nonsmall cell lung cancer or limited disease for small cell lung cancer. For completeness, we also examined the proportion with advanced lung cancer (*post hoc*), which might be less influenced by overdiagnosis. Other end-points included: uptake of sputum sampling, AFB and LDCT; proportion of participants in the surveillance arm with abnormal sputum cytology and/or cytometry; number of failed/inadequate sputum samples; and prevalence of pre-invasive disease among participants with abnormal cytometry/cytology.

The proportion of individuals with lung cancer who were diagnosed at an early (or advanced) stage was compared between the trial arms (relative risk) and also rate ratio using person-years. Additional analyses were performed to check consistency in the findings. Estimates of screening performance for each test separately were: 1) sensitivity (proportion of all lung cancers with positive test results) and 2) FPR (proportion of all those without lung cancer with positive test results).

### Statistical methods

15% of controls were expected to be diagnosed at an early stage [34]. From prior LDCT studies and our pilot study of pre-invasive disease, 80% of cancers were stage I/II [29], so we conservatively used 50%. To detect a difference of 15% *versus* 50% required a target sample size of at least 37 lung cancers per arm (95% power and 5% one-sided significance test pre-specified for this preliminary study). The expected total proportion of prevalent and incident lung cancers was ∼6% [9], so to obtain 74 cancers required about 1700 individuals.

## Results

**FIGURE 1:**
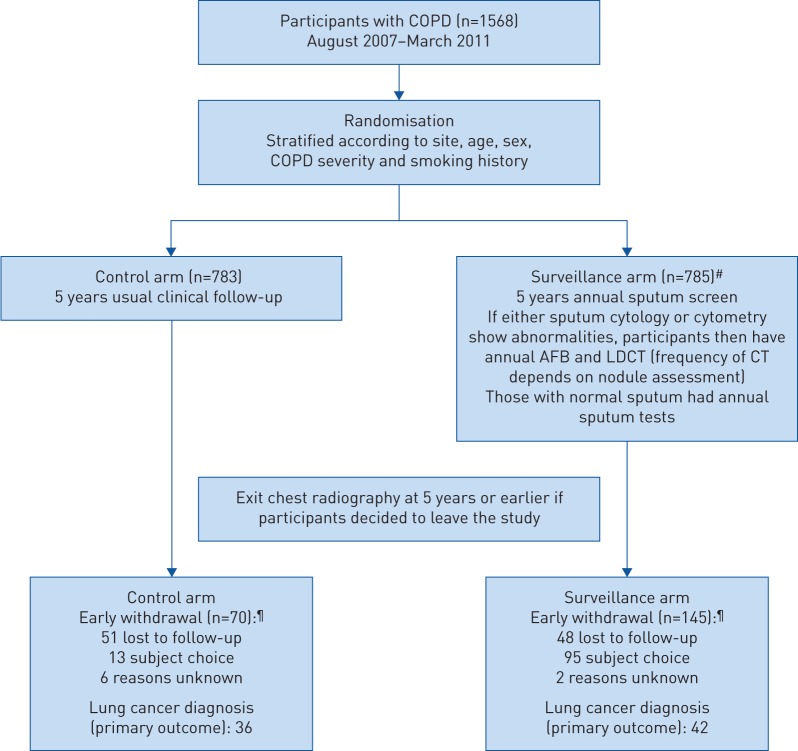
CONSORT diagram. COPD: chronic obstructive pulmonary disease; AFB: autofluorescence bronchoscopy; LDCT: low-dose computed tomography. Supplementary table S1 provides further details about number of participants approached and trial uptake. ^#^: it transpired that one person actually had lung cancer >1 year prior to randomisation but did not inform the trial staff (they would have been ineligible). Because this was only discovered at the end of the trial (cancer notification by the national registry), the person was kept in the intention-to-screen analyses. The person had normal sputum samples throughout and no AFB or CT (and not counted as a cancer case). Counting this as a cancer case had only a small effect on sensitivity (44.7% without it (figure 2) and 43.6% with it). ^¶^: even though some participants withdrew from the trial procedures before 5 years, they were still flagged for cancer occurrence.

1568 participants (785 screened and 783 controls) were recruited from 10 UK centres between August 2007 and March 2011 (figure 1 and supplementary table S1). Baseline characteristics were balanced (table 1).

**TABLE 1:** Baseline characteristics of the randomised individuals

	**Controls**	**Screened**
**Participants**	783	785
**Sex**		
Female	373 (48)	377 (48)
Male	410 (52)	408 (52)
**Smoking status**		
Current smoker	435 (56)	439 (56)
Ex-smoker	348 (44)	346 (44)
**COPD severity**		
Mild	195 (25)	196 (25)
Moderate	588 (75)	588 (75)
* *Missing/unknown	0	1
**Source of participants**		
General practice	622 (79)	619 (79)
Pulmonary rehabilitation programme	95 (12)	94 (12)
Hospital outpatients	35 (4)	42 (5)
Lung function laboratory	31 (4)	30 (4)
**Mean age at randomisation years**	63	63
**Mean age when started smoking years**	16	16
**Mean age when stopped smoking years**	61 (n=348)	62 (n=346)
**Mean cigarettes smoked per day n**	24	24
**Mean smoking duration years**	45	45
**Mean pack-years**	53	54

Data are presented as n or n (%). COPD: chronic obstructive pulmonary disease.

Seven centres routinely collected screening logs of individuals approached: 38.7% of all those contacted by telephone after the initial search accepted the invitation to attend the pre-trial assessment, of which 42.4% were randomised (supplementary table S2). The initial uptake (38.7%) was high compared with LDCT screening trials, and probably due to our focus on COPD patients who might be more aware of smoking-related risks and their chronic symptoms influenced their decision to enrol, compared with a more general population. Older individuals were more likely to decline to participate in the trial (OR 1.92 for ≥70 *versus* <50 years; p<0.0001). There was no association with sex, but there were geographical differences (supplementary table S3).

### Provision of sputum samples

In the first year (baseline), 89.8% provided sputum samples, but 36 were inadequate for assessment (so 85.2% provided an evaluable sample). Of those with adequate samples, 19.0% were abnormal for either cytology or cytometry and the rate was lower in subsequent years (table 2). The percentage not providing an adequate sputum sample increased from 14.8% at baseline up to 46.1% by year 5.

**TABLE 2:** Sputum results in the screened group in each year

	**Baseline**	**Year 2**	**Year 3**	**Year 4**	**Year 5**
**Cytology or cytometry result**^#^	785^¶^	639^¶^	560^¶^	516^¶^	447^¶^
Normal	542 (81)	398 (87)	343 (94)	300 (89)	221 (92)
Abnormal					
Low grade	111 (17)	51 (11)	18 (5)	33 (10)	17 (7)
High grade	16 (2)	6 (1)	2 (1)	4 (1)	3 (1)
Died or cancer diagnosed since last visit		19	22	24	32
No sputum result	116 (15)^+^	184 (29)^+^	197 (35)^+^	179 (35)^+^	206 (46)^+^
Did not provide sample	68	131	155	157	195
Tried but unable to provide sample	12	10	3	8	5
Provided spontaneous sample^§^	33	43	38	14	6
Provided induced sample^§^	3	0	1	0	0
**Cytology result (where available)**	604	400	301	285	198
Normal	503 (83)	358 (90)	289 (96)	269 (94)	191 (96)
Abnormal					
Low grade	86 (14)	36 (9)	11 (4)	13 (5)	5 (3)
High grade	15 (2)	6 (2)	1 (<1)	3 (1)	2 (1)
**Cytometry result (where available)**	603	418	350	323	237
Normal	570 (95)	400 (96)	342 (98)	298 (92)	221 (93)
Abnormal					
Low grade	32 (5)	18 (4)	7 (2)	22 (7)	15 (6)
High grade	1 (<1)	0	1 (<1)	3 (1)	1 (<1)

Data are presented as n or n (%); the percentages in brackets for normal or abnormal sputum are based on the total number who had a sputum result as the denominators. ^#^: in some cases only cytology or cytometry results were available (not both) and so the result classification was based on the known result if a repeat sputum sample was not done; ^¶^: total number of individuals expected to provide sputum samples in each year (*i.e.* excluding those who had an abnormal sputum result, died or were diagnosed with cancer who were no longer expected to provide sputum samples); ^+^: the percentage who did not provide a sputum sample, out of the total expected; ^§^: sample was inadequate for cytology and cytometry assessment.

33.2% of all individuals in the screened arm had an abnormal sputum result at any time, of which 22.5% had abnormal cytology and 12.6% had abnormal cytometry (1.9% (15 out of 785) had both abnormal cytology and cytometry, 20.6% (162 out of 785) had abnormal cytology only, and 10.7% (84 out of 785) had abnormal cytometry only). 82.4% (14 out of 17) of sputum-positive cancers were detected at an early stage compared with 38.1% (eight out of 21) of sputum-negative cancers (p=0.01). Cytology, which used morphological criteria alone, identified more cancers than image cytometry (12 *versus* five) among those with abnormal sputum, so they appeared to be complementary. No cancer had both abnormal cytology and cytometry. There was no discernible association between type of sputum test and histology, particularly with having only few cases.

### Primary end-point

78 lung cancers were identified (36 and 42 in the control and screened groups, respectively); the Kaplan–Meier plot is given in supplementary figure S2. The median follow-up was 5 years, matching the planned duration in the protocol for each participant.

Table 3 shows histology and cancer staging. Overall, 54.8% of screened individuals *versus* 45.2% of controls, with known staging, were diagnosed at an early stage (similar to 59.4% *versus* 48.1% for nonsmall cell lung cancer alone). Table 4 compares stage at diagnosis between the trial arms. The relative risk for early-stage cancer detection was 1.21 (95% CI 0.75–1.95; one-sided exact p=0.24) or 0.82 (95% CI 0.52–1.31) for advanced cancers. Hence, there was no clear stage shift. In the sensitivity analyses, the rate ratio was a secondary analysis (not pre-specified in the trial protocol) and although the estimate for early-stage disease made screening appear favourable (1.83, 95% CI 0.94–3.54), there was no corresponding reduction in advanced cancers (1.24, 95% CI 0.65–2.39). Furthermore, the size of the absolute difference in stage (either early or advanced) is not clinically important.

**TABLE 3:** Histology and stage of the lung cancers

	**Controls**	**Screened**
**Cancers**	36	42
**Small cell**	5 (14)	10 (24)
**Adenocarcinoma**	8 (22)	11 (26)
**Squamous**	9 (25)	14 (33)
**Large cell**	0	1 (2)
**Other histology**	9 (25)	5 (12)
**Unknown**	5 (14)^#^	1 (2)
**Nonsmall cell lung cancer**	27^¶^	32^¶^
Stage I	11	16
Stage II	2	3
Stage III	6	4
Stage IV	7	9
Unknown	1	
**Small cell lung cancer**	5	10
Limited disease	1	4
Extensive disease	4	6

Data are presented as n or n (%). The exit chest radiography found five cancers in the screened group (these had no sputum samples or their sputum tests were normal throughout the trial: cancer stage was I (n=2), II (n=1), IV (n=1) and limited disease (n=1)) and six cancers in the control group (stage was I (n=3), III (n=1), IV (n=1) and missing (n=1)). ^#^: diagnosed at nontrial sites (unknown or not set up for the trial so no access to medical records; these cancers were notified through registries and we found staging for one of the five cases); ^¶^: includes one patient in each trial group where histology was unknown but stage was available.

**TABLE 4:** Comparison of stage at diagnosis among those with lung cancer (in total there were 42 and 36 lung cancers in the screened and control arms, respectively)

	**Early-stage disease (I/II for nonsmall cell cancer and limited disease for small cell cancer) (primary outcome measure)**		**Advanced disease (III/IV for nonsmall cell cancer and extensive disease for small cell cancer)**	
	**Screened**	**Controls**	**Screened**	**Controls**
**Main analysis (cancer cases with known stage)**	54.8% (23/42)	45.2% (14/31)	45.2% (19/42)	54.8% (17/31)
	Relative risk 1.21 (95% CI 0.75–1.95; p=0.24)		Relative risk 0.82 (95% CI 0.52–1.31; p=0.24)	
**Sensitivity analyses**				
All cancers included in the denominators	54.8% (23/42)	38.9% (14/36)	45.2% (19/42)	47.2% (17/36)
	Relative risk 1.41 (95% CI 0.86–2.30; p=0.09)		Relative risk 0.96 (95% CI 0.59–1.55; p=0.50)	
Excluding cancers found by exit chest radiography (n=5 screened; n=6 controls)	51.3% (19/37)	42.3% (11/26)	48.9% (18/37)	57.8% (15/26)
	Relative risk 1.21 (95% CI 0.70–2.09; p=0.30)		Relative risk 0.84 (95% CI 0.53–1.35; p=0.30)	
Cancer incidence expressed as person-years	6.8 per 1000	3.7 per 1000	5.6 per 1000	4.5 per 1000
	Rate ratio 1.83 (95% CI 0.94–3.54; p=0.049)		Rate ratio 1.24 (95% CI 0.65–2.39; p=0.31)	
Cancer incidence expressed as person-years and excluding cancers found by exit chest radiography	5.7 per 1000	3.0 per 1000	5.4 per 1000	4.0 per 1000
	Rate ratio 1.92 (95% CI 0.91–4.03; p=0.049)		Rate ratio 1.33 (95% CI 0.67–2.64; p=0.24)	

Relative risk or rate ratio of >1 for early stage indicates that screening was effective (more early-stage disease found in the screened group). Relative risk or rate ratio of <1 for advanced stage indicates that screening was effective (less advanced-stage disease found in the screened group). Rate ratio, which uses person-years, might be less affected by overdiagnosis and unknown disease stage in the denominators. All p-values are one-sided (specified in the protocol) because of interest only in finding more early-stage cancers in the screened arm. LungSEARCH is not a definitive assessment of a screening policy, so it is analogous to phase II treatment trials that commonly use one-sided statistical tests.

### Screening performance

Table 5 summarises the findings of all three tests among the lung cancers in the screened group: 44.7% had an abnormal sputum sample, but 55.3% (21 cases) had normal results for all samples.

**TABLE 5:** Test findings among all 42 lung cancers in the screened group

**Sputum result**	38
Abnormal	17 (45)
Normal	21 (55)
No sputum or both cytology/cytometry inadequate	4
**Cytology result**	38
Abnormal	12 (32)
Normal	26 (68)
**Cytometry result**	38
Abnormal	5 (13)
Normal	33 (87)
**Worst AFB result**	11
Carcinoma	2 (18)
Moderate dysplasia	2 (18)
Squamous metaplasia	1 (9)
No abnormality	6 (55)
**Sputum and LDCT results**	42
No sputum samples (hence no LDCT)	4 (2)^#^
Sputum normal throughout study (hence no LDCT)	21 (3)^#^
Sputum abnormal, LDCT detected cancer directly afterwards^¶^	8
Sputum abnormal, LDCT detected cancer at a later follow-up^+^	7
Sputum abnormal, LDCT did not flag for cancer investigation^§^	1
Sputum abnormal, but no LDCT done	1

Data are presented as n or n (%), unless otherwise stated. AFB: autofluorescence bronchoscopy; LDCT: low-dose computed tomography. ^#^: the numbers in brackets are lung cancers found by the exit chest radiography at 5 years; ^¶^: the abnormal sputum result led directly to an abnormal CT (*i.e.* a nodule ≥9 mm) and the individuals were referred for immediate diagnostic investigations; ^+^: individuals had an abnormal sputum and the abnormal CT that found the cancer was one of the later follow-up scans (in three cases, the first CT with a nodule ≥9 mm was some years before the cancer diagnosis but subsequent CT scans indicated that the nodule had shrunk before the final CT that led to diagnostic investigations showed nodule growth); ^§^: the individual had normal annual CT scans during the trial (the cancer was found by a CT scan given outside of the protocol when the person finished the study; a suspicious large nodule ≥9 mm had appeared).

**FIGURE 2:**
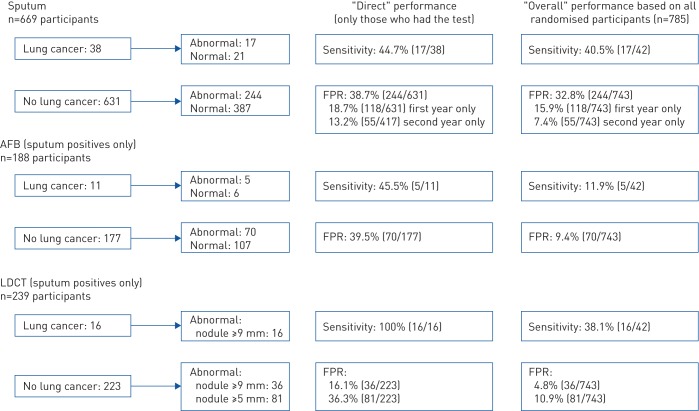
Summary of screening performance for the three tests in the surveillance group based on results at any time during the trial. FPR: false-positive rate; AFB: autofluorescence bronchoscopy; LDCT: low-dose computed tomography. Sensitivity indicates percentage of cancers with abnormal results. FPR indicates percentage of individuals without lung cancer with abnormal results (same as 1−specificity).

Figure 2 summarises sensitivity and FPR for all three tests estimated only among individuals who actually had the tests (labelled “direct”) and among all 785 individuals randomised to surveillance (labelled “overall”) (see further description in the supplementary material). The measures for LDCT and AFB can only be interpreted in the context of being second-stage tests, and do not represent performance for population screening where everyone has the test(s).

In the screened group, the overall sensitivity for sputum was 40.5% and FPR was 32.8%. When examining only those who had sputum results, the direct sensitivity for cytology/cytometry was 44.7% and the corresponding FPR was 38.7% (figure 2). Hence, sputum testing did not detect many cases. The direct FPR at baseline only was 18.7% and was lower in the subsequent year at 13.2%. Sputum testing had insufficient screening performance.

188 individuals had an AFB at any time during the trial (an additional 73 declined or did not attend; uptake 72.0%). Only 11 sputum-positive cancer cases had AFB and the direct sensitivity was 45.5%, with a high FPR of 39.5% (figure 2). Among participants with abnormal sputum, 38% had pre-invasive disease (72 out of 188 mild to severe dysplasia or metaplasia); only three of these (two moderate dysplasia and one squamous metaplasia) later developed lung cancer.

239 individuals had LDCT at any time during the trial (an additional 22 declined or did not attend; uptake 91.6%). 16 sputum-positive cancer cases had LDCT and the direct sensitivity (nodule size ≥9 mm) was 100%, with a FPR of 16.1% (figure 2).

### Other cancers, mortality and smoking status

Supplementary table S4 summarises the end of trial status, including the number who had an exit chest radiograph (430 screened and 486 controls, a difference that is unlikely to have materially biased the cancers found). Other cancer types were balanced between the two groups. Lung cancer mortality (16 screened *versus* 21 controls; hazard ratio 0.86; p=0.65), and all-cause mortality (hazard ratio 0.87; p=0.39) were similar (supplementary figure S3). Among those who were current smokers at baseline (with known smoking status at 5 years), 15.0% of controls and 17.7% of screened individuals had stopped completely during the trial.

### Adverse events

In the surveillance group, one person had a COPD exacerbation possibly linked to AFB and another committed suicide unrelated to study participation.

## Discussion

We examined a sequential approach to only offer LDCT and AFB as second screening tests among particularly high-risk individuals with abnormal sputum cytology/cytometry. Had we found a substantial stage shift, a larger randomised trial of lung cancer mortality would overcome lead-time bias and overdiagnosis. LungSEARCH complements LDCT trials [2, 6], including the only other randomised trial of lung cancer screening conducted in the UK (the UK Lung Cancer Screening Trial) [33].

Although LungSEARCH preceded NLST and NELSON [5, 6], the concept that an effective, cheap and easy initial test (sputum) could be considered for a wider group of smokers than is currently eligible for LDCT remains valid. This is because current criteria exclude many high-risk individuals. Applying US Preventive Services Task Force criteria [7], 25% of the LungSEARCH participants would be ineligible for LDCT. We hoped, therefore, that our sequential approach could find many cancers without offering many more LDCT scans.

We exceeded the target of 50% of lung cancers diagnosed at an early stage using our surveillance strategy (observed 55%), but the lack of effect was driven by the high percentage of unscreened participants diagnosed at early stage (45% observed instead of 15% expected when LungSEARCH was designed in 2006). Prominent health campaigns have encouraged individuals with persistent cough to seek medical attention sooner, explaining why more lung cancers are now diagnosed earlier, as seen in UK audit data [35]. Although we reached the target sample size and hence had power for the expected primary outcome (50% *versus* 15% early-stage cancers), the observed small stage shift of 55% *versus* 45% is not worthwhile clinically.

In LungSEARCH, 90% of those who attempted a sputum sample at baseline did so successfully. However, an increasing number of individuals did not provide sputum over time and four lung cancers were among participants who provided no samples. Hence, 60% of all lung cancers in the screened group did not have the opportunity for earlier detection by LDCT. Furthermore, of the cancers with sputum samples, only 45% had abnormal results (referred for LDCT and AFB). This is lower than the expected 80% from a study that had more males than LungSEARCH and 59% had moderate/severe cough, although in that study the sensitivity of sputum decreased to 21% for stage I adenocarcinoma [21]. It is unclear why sputum was not effective. Unlike cervical cancer screening, which involves active removal of cells in the cervix, detecting lung cancer in sputum depends on cells naturally shed into the bronchi, which is influenced by tumour location and histology. It could be that malignant cells in the early stages of lung cancer are still anchored to the basement membrane and each other, so that not enough travel into the lumen. Although sputum testing has the appeal of being conducted at home, avoiding travel to screening clinics which is required by LDCT (especially from rural areas), the lower number of individuals who provided samples from year 2 plus the fact that several samples were inadequate together makes sputum testing less useful than LDCT, in which a result could be obtained in almost all cases who are scanned.

AFB uptake was not high (72%), because several participants informed us that they found AFB off-putting or uncomfortable [36]. Systematic reviews of AFB show heterogeneous study designs and variable sensitivities (67–100%) [37–39]. While AFB has value for individuals presenting with symptomatic lung problems, LungSEARCH suggests a limited role in screening. Improvements in the optics in videobronchoscopes have also reduced the need for the fluorescence mode and the shift in the natural history of lung cancer from central to more peripheral tumours further limits the utility of AFB.

Very few reports have examined lung cancer screening in COPD. The NLST substudy (in the NLST American College of Radiology Imaging Network (ACRIN) cohort) indicated a shift towards early-stage cancer among COPD participants who had LDCT compared with those who had chest radiography [40], but no reduction in lung cancer deaths [41]. The Danish Lung Cancer Screening Trial hinted that COPD participants with >35 pack-years might benefit from LDCT [42], whereas in a nonrandomised matched cohort study of mild/moderate COPD, 80% of lung cancers in those who had LDCT were diagnosed at stage I *versus* 0% among those without LDCT, with corresponding lung cancer deaths of one *versus* 12 (p=0.002) [43].

Our trial had limitations. As in all cancer screening trials, participants could not be blinded, hence the potential for bias (*e.g.* controls were aware of the trial objectives possibly making them more alert to symptoms and seeking medical advice sooner), which might contribute to the higher than expected proportion of early-stage cancers. Similarly, participants who stopped having the screening tests earlier might lead to a lower percentage diagnosed with early-stage cancer. We had no data on cancer treatments nor retrieved histological specimens for central pathology review, as these required additional local resources. Overdiagnosis bias is an established issue in studies examining stage shift. We found slightly more lung cancers in the screened group (n=42) than controls (n=36) and the different denominators (expected in screening studies) can influence the comparison of stage shift. Therefore, we allowed longer time for cancer notifications from the registries and to arrange the exit chest radiographs in the controls. Although we did not find a material difference in cancer stage in LungSEARCH, there is some evidence that individuals with COPD tend to develop more aggressive lung cancers [44, 45]. The NLST trial suggests that overdiagnosis from LDCT screening is only seen in individuals with normal lung function, not in COPD, although this should be confirmed in other studies [40]. Finally, we did not know whether some of the control group participants had LDCT during the trial, which might have reduced the effect of our screening policy, although we expect this to be very few because LDCT is not recommended routinely.

LDCT screening can be made more efficient using risk algorithms (including age and smoking intensity), where only those with a risk exceeding a specified cut-off are offered LDCT. Such models detect more lung cancers with fewer false positives than current criteria [7]. Several risk calculators contain COPD as a factor [46–48], and demonstration/pilot studies in the UK conclude that the Liverpool Lung Project risk model and/or the PLCO_M2012_ model (from the Prostate, Lung, Colorectal and Ovarian (PLCO) Cancer Screening Trial) should be used to identify a high-risk population in screening programmes [49–52]. These recommendations are supported by LungSEARCH in which LDCT detected all lung cancers among sputum positives (although we cannot tell how well LDCT would have performed in the sputum-negative cases and our trial did not include individuals without COPD).

In conclusion, our sequential screening strategy did not show a stage shift in cancer diagnosis. Our trial has implications for future research and practice. First, it provides evidence from a large randomised trial that it is difficult to find ways of targeting LDCT screening to make it more efficient (other than risk-based algorithms). LDCT should therefore be offered to all eligible individuals within planned screening programmes. Second, our study was based on particularly high-risk individuals (smokers with COPD) and many unscreened individuals (controls) were diagnosed at an early cancer stage, indicative of them seeking medical attention sooner. This probably means that this group is more receptive to screening and early detection than previously thought, such that the uptake of LDCT within organised programmes could be high among these individuals. Third, LDCT detected all lung cancers among COPD patients in our trial who were sputum positive, which is suggestive evidence that planned screening programmes should consider sufficient inclusion of COPD.

## Supplementary material

10.1183/13993003.00581-2019.Supp1 **Please note:** supplementary material is not edited by the Editorial Office, and is uploaded as it has been supplied by the author.
Supplementary material ERJ-00581-2019.SUPPLEMENT

## Shareable PDF

10.1183/13993003.00581-2019.Shareable1 This one-page PDF can be shared freely online.
Shareable PDF ERJ-00581-2019.Shareable
