# Supplementary material for: Sequential screening for lung cancer in a high-risk group: randomised controlled trial: LungSEARCH: a randomised controlled trial of Surveillance using sputum and imaging for the EARly detection of lung Cancer in a High-risk group
Source: Eur Respir J. 2019 Oct 17;54(4):1900581. doi: 10.1183/13993003.00581-2019 (PMC6796151; doi:10.1183/13993003.00581-2019)
Supplement: Supplementary file 1 [file ERJ-00581-2019.SUPPLEMENT.pdf]

Supplemental Appendix

Appendix Text 1	Further details and methods of the sputum sampling
Appendix Text 2	Further details of auto-florescence bronchoscopy
Appendix Text 3	Further details of the low dose CT scan (LDCT)
Appendix Text 4	Description of screening performance for the 3 tests used (sputum, AFB and LDCT)
Appendix Table S1	Distribution of trial participants across the 10 centres
Appendix Table S2	Summary of recruitment activity among the 7 centres that had screening logs
Appendix Table S3	The odds ratio (95% CI) of declining to participate in LungSEARCH according to geographical location (centre), age and sex
Appendix Table S4	End of trial status, including the exit chest radiography
Appendix Figure S1	Flow diagram for trial participants in the screened arm
Appendix Figure S2	Kaplan-Meier plot for the incidence of lung cancer
Appendix Figure S3	Kaplan-Meier plots for deaths due to lung cancer (upper), and all-cause mortality (lower)

Appendix Text 1. Further details of the sputum sampling

Individuals in the screened group provided annual sputum samples (three pots) at home. Samples were posted to the central laboratory at University College London Hospital, who prepared four slides using Thin-Prep-2000 processor (Cytyc UK). Two had Papanicolaou staining[a] for cytology review, and two were stained with a modified Feulgen's reagent[b] for cytometry.

- Cytology samples were considered assessable if each contained ≥5 alveolar macrophages and/or bronchial cells. Morphological parameters were graded using the Bethesda classification system[c] for Squamous Intraepithelial Lesions (SIL). The presence of any atypia, either low- or high-grade SIL was considered 'abnormal'.
- Cytometry: a semi-automated system was used (Fairfield DNA ploidy, Fairfield Imaging, Nottingham, UK), in which DNA histograms were examined and samples classified as having normal or abnormal DNA contents using published criteria.[d]

a. Papanicolaou GN. A new procedure for staining vaginal smears. Science. 1942; 95: 438–439.

b. Schulte E, Wittekind DH. Standarization of the Feulgen reaction: the influence of chromatin condensation on the kinetics of acid hydrolysis. Annal Cell Pathol 1990; 2:149–157.

c. Solomon D, Nayar R. The Bethesda System for Reporting Cervical Cytology. Definitions, Critera and Explanatory Notes., 2nd ed. Springer Verlag, New York;2004:1-191.

d. Sudbo J, Kildal W, Risberg B, Koppang HS, Danielsen HE, Reith A. DNA content as a prognostic marker in patients with oral leukoplakia. N Engl J Med. 2001;344:1270-8.

Appendix Text 2. Further details of auto-florescence bronchoscopy (AFB)

Delivery of AFB

AFB was performed under conscious sedation, and the bronchial tree examined under different lights using optical filters incorporated into the bronchoscope (D-light auto-fluorescence system, Karl Storz Gmbh, Germany; or auto-fluorescence video-bronchoscope, Olympus Medical, Japan). If AFB appeared abnormal under either white or blue light, 1-3 bronchial biopsies (for histology review) were taken from each affected area, and also an area with normal appearance on the contralateral side. For individuals without an abnormality, three biopsies were taken from a single area of normal appearance. Specimens were reviewed locally by an expert pulmonary histopathologist, categorized as squamous metaplasia, mild to severe dysplasia, carcinoma *in situ* or carcinoma.

Categories of dysplasia using AFB and subsequent histopathology:
1 = Squamous metaplasia
2 = Mild dysplasia
3 = Moderate dysplasia
4 = Severe dysplasia
5 = Carcinoma-in-situ
6 = Carcinoma

If no pre-invasive lesion were found after histopathology review of the bronchial biopsy tissue samples, AFB was repeated annually.

If a pre-invasive lesion were found by histopathology, AFB was repeated at intervals according to the grade of pre-invasive lesion:

- for carcinoma in situ and severe dysplasia (categories 4-5) the interval could be approximately 5 months
- for moderate to mild dysplasia (categories 2-3) the interval would be approximately 8 months.

If an invasive lesion (category 6) were found the individual was referred for other investigations and treatment via the normal hospital systems.

Appendix Text 3. Further details of the low dose CT scan (LDCT)

LDCT (target radiation dose <2mSv) was conducted without contrast, and assessment of nodules largely determined the frequency of subsequent follow-up using LDCT. Suspicion of cancer (a nodule size ≥9mm) could lead directly to CT with contrast, PET/CT or other investigations for cancer according to local practice.

<u>LDCT delivery</u>

No Intravenous contrast for initial scan

- Width section needs to be 1mm or equivalent with a multi-detector row CT scanner
- Axial +/- coronal reformats if available [Review MIPs from work stations if available]
- Low dose CT equivalent [depending on local practice /Dose modulation CT packages available, and patient's habitus]. Standard dose for CT scan if the nodule demonstrates growth or suspicious features with IV contrast.
- Images viewed from computer workstations with standard lung / soft tissue and bone settings.

<u>Assessment of Nodules</u>

- Document for non-calcified nodules
  1. Anatomical site
  2. Size-Volume assessment with maximum diameter all three planes. Growth of >25% is considered significant and further follow-up required
  3. Morphology:
     - Round or oval
     - Smooth or irregular margin
     - Solid or ground glass / semisolid
     - +/- cavitation
     - calcific foci

Those with mass lesions suspicious for lung cancer underwent urgent investigations as deemed appropriate by their clinician. Indeterminate non-calcified nodules were to be followed up according to their size as reported by references a-d below:

Nodule size	Recommended frequency of CT scan	
< 5 mm	Annually	Follow-up non contrast-enhanced CT, to look for growth.
≥ 5 to < 7 mm	At 6, 12 and 18-24 months	Follow-up non contrast-enhanced CT. If growth assess with IV contrast.
≥ 7 to < 9 mm	At 4, 8, 12 and 24 months	Follow-up non contrast-enhanced CT. If growth assess with IV contrast.
≥ 9 mm	(a) If nodule appears benign: CT at 4, 8, 12 and 24 months<br><br>(b) If nodule appears malignant: CT with contrast	Follow-up (IV) contrast-enhanced CT.<br><br>For malignant-looking nodules: investigate for cancer with dynamic CT, PET/CT, biopsy, FNA, or surgery, as indicated by local practice.

a. Henschke CI, McCauley DI, Yankelvitz DF et al. Early detection lung project: overall design and findings from baseline screening. Lancet 1999;354:99-105.
b. Swensen SJ, MD, Jett JR, Sloan JA, et al. Screening for lung Cancer with low dose helical computed tomography. Am J Respir Crit Care Med. 2002; 165:508-513.
c. Pastorino U, Bellomi M, Landoni C, et al., Early lung-cancer detection with spiral CT and positron emission tomography in heavy smokers: 2-year results Lancet 2003;362:593–97.
d. Fleischner society, Radiology 237, 2,395-400 2005

Central quality control audit

Two chest radiologists at UCLH (not managing trial patients) conducted independent quality assurance audits between April 2009 and July 2014. Double blind reviews of randomly selected LDCT scans retrieved from all sites were carried out, and the LDCT case report forms (CRFs) were also audited. Early in the trial, double reporting of scans at each site was undertaken, contributing to delays in sending the data to the trials centre. The independent review confirmed a high concordance between local reports, so that single reporting was implemented for the remainder of the trial (which consequently improved CRF return). The central review demonstrated a delay between a positive sputum result and having a LDCT in some cases, successfully leading to a change in practice which minimized/avoided the delay. The central review resulted in the same outcomes of the scans (i.e. cancer referral or timing of next follow-up scan) as the local assessment in the majority (97%) of cases.

Appendix Text 4. Description of screening performance for the 3 tests used (sputum, AFB and LDCT)

33.2% (261/785) of all individuals in the screened arm had an abnormal sputum result at any time, of which 22.5% (177/785) had abnormal cytology, and 12.6% (99/785) had abnormal cytometry. Among these 261, only 15 had both abnormal cytology and cytometry (162 abnormal cytology alone and 84 abnormal cytometry alone). 38 of all lung cancers in the screened group had sputum results, and 17 were abnormal at some point: 12 abnormal cytology alone and 5 abnormal cytometry alone (Table 5).

Table 5 shows that 21 lung cancer cases had a normal sputum throughout and were diagnosed outwith the trial procedures (4 adenocarcinoma, 5 squamous, 8 small cell, 1 large cell, and 3 other types). 8 (38.1%) were at an early stage, much lower than among the 17 cases that had an abnormal sputum, where 14 were diagnosed at an early stage (82.4%) and all were found by LDCT. Among the 3 cases with abnormal sputum diagnosed at late stage, two were found by LDCT directly following the abnormal sputum result, and the other case had neither an AFB nor LDCT.

No cancer had both abnormal cytology and cytometry. There was no discernable association between type of sputum test and histology, particularly with having only few cases.

When examining only those who had sputum results, the direct sensitivity was 44.7% (17/38), and corresponding FPR 38.7% (244/631); Figure 2. When considering all 42 lung cancers found and all 743 individuals without lung cancer, the overall sensitivity was 40.5%, and FPR 32.8%. These are cumulative FPRs by year 5. The direct FPR at baseline only was 18.7% (118/631) and in the subsequent year only it was 13.2% (55/417).

188 individuals had an AFB at any time during the trial (an additional 74 declined or did not attend; uptake 71.7% [188/188+74]). Of these, 39.9% (75/188) were abnormal (metaplasia, dysplasia, carcinoma or carcinoma *in situ*). The overall prevalence of pre-invasive disease among participants with abnormal sputum was 38% (72/188 had mild to severe dysplasia or metaplasia; but only 3 of these [2 moderate dysplasia and 1 squamous metaplasia], later developed lung cancer, 2.3-10.1 months later). Of the 17 lung cancers who had abnormal sputum, 6 never had AFB; whilst 11 did, of which 5 had suspicious lesions/dysplasia: direct sensitivity 45.4% and FPR 39.5% (Figure 2). Two of these 5 were confirmed as cancer after histopathology review (Table 5), where one was visualized by AFB on the right main bronchus (missing information for the other). For two other cases, AFB appeared normal but histopathology of the biopsy taken exhibited dysplasia.

239 (30.4%) individuals had a LDCT at any time during the trial (an additional 22 declined or did not attend; uptake 91.6% [239/239+22]). Of these, 21.8% (52/239) had at least one nodule of ≥9mm, considered for immediate diagnostic investigation. 18.8% (45/239) had nodules between 5 and 9mm, requiring LDCT scans more regularly than annually, but no immediate cancer investigations, and none of these 45 were diagnosed with lung cancer during the study. Among all 42 lung cancers, 16 had a LDCT (Table 5), of which 15 had an abnormal finding (nodule ≥9mm) during the trial and then referred for cancer diagnoses, and the other case was found by LDCT performed at trial exit (a nodule ≥9mm). Figure 2 shows that the direct sensitivity was 100%; and FPR 16.1% (36/223, using nodule size ≥9mm) or 36.3% (81/223, using ≥5mm).

Appendix Table S1. Distribution of trial participants across the 10 centres.

	N=1568
Chelsea & Westminster	348 (22%)
Cambridge	301 (19%)
University College Hospital London	277 (18%)
Leeds	206 (13%)
Belfast	106 (7%)
Leicester	89 (6%)
Royal Brompton	75 (5%)
Manchester	65 (4%)
Coventry	61 (4%)
Sunderland	40 (3%)

Appendix Table S2. Summary of recruitment activity among the 7 centres that had screening logs

	Number contacted	Number who did not reply	Number who replied to invitation		Number who were randomized#
			Declined to participate	Accepted	
UCH	1580	400 (25%)	470 (30%)	710 (45%)	277
Brompton	225	67 (30%)	39 (17%)	119 (53%)	75
Chelsea & Westminster					
Hospitals	64	5 (8%)	19 (30%)	40 (62%)	4
GPs	2437	368* (15%)	1003* (41%)	1066* (44%)	344
Cambridge	1368	212 (15%)	738 (54%)	418 (31%)	301
Leeds	1622	362 (22%)	709 (44%)	551 (34%)	206
Belfast	702	343 (49%)	164 (23%)	195 (28%)	106
Total	7998	1757 (23%)	3142 (39%)	3099 (39%)	1313**

*approximate

** 1568 in total in the trial

out of those who accepted the invitation and were eligible after baseline tests

39% (3099/7998) of all those contacted by telephone after the initial search accepted the invitation to attend the pre-trial assessment, of which 42% (1313/3099) were randomized.

Appendix Table S3. The odds ratio (95% CI) of declining to participate in LungSEARCH according to geographical location (centre), age and sex.

Factor		Univariable (based on all available data for the factors)	Univariable (only subjects with non-missing data for all 3 factors)	Multivariable (only subjects with non-missing data for all 3 factors)*
**Location:**				
No. subjects		4327	3747	3747
No. who declined		2974	2394	2394
UCH		1.0	1.0	1.0
Brompton		0.83 (0.56-1.23)	0.31 (0.16-0.58)	0.29 (0.15-0.55)
Chelsea & Westminster		1.07 (0.85-1.35)	0.76 (0.57-1.00)	0.74 (0.56-0.98)
Cambridge		3.65 (2.94-4.54)	5.71 (4.49-7.26)	5.41 (4.25-6.90)
Leeds		5.94 (4.72-7.48)	9.57 (7.45-12.30)	9.63 (7.47-12.41)
Belfast		1.27 (0.93-1.75)	2.01 (1.44-2.81)	2.01 (1.43-2.81)
Sunderland		9.52 (6.56-13.81)	3.41 (2.21-5.28)	3.63 (2.34-5.64)
		P<0.0001	P<0.0001	P<0.0001
**Age:**				
No. subjects		3755	3747	3747
No. who declined		2402	2394	2394
Age	<50 years	1.0	1.0	1.0
	50-59	1.95 (1.47-2.59)	1.96 (1.48-2.60)	1.27 (0.92-1.76)
	60-69	1.84 (1.41-2.39)	1.85 (1.42-2.41)	1.22 (0.90-1.65)
	70+	2.53 (1.92-3.34)	2.54 (1.93-3.35)	1.92 (1.40-2.63)
		P<0.0001	P<0.0001	P<0.0001
**Sex:**				
No. subjects		4300	3747	3747
No. who declined		2947	2394	2394
	Males	1.0	1.0	1.0
	Females	1.10 (0.96-1.25)	1.13 (0.99-1.29)	1.02 (0.88-1.19)
		P=0.16	P=0.08	P=0.80

*Odds ratios are adjusted for the other two factors in the table

Univariable and multivariable logistic regressions were used to examine the odds of declining to participate (adjusted for age, sex and geographical location). This information could be used to consider potential factors that might influence future lung screening uptake in the UK (acknowledging that here, people were asked for participation in a randomized study of screening, rather than screening per se). People from Belfast, Cambridge, Leeds and Sunderland were more likely to decline than those from the University College London Hospital area (odds ratios of 2.01, 5.41, 9.63 and 3.63 respectively), while those from the Brompton Hospital and Chelsea and Westminster areas were less likely to decline (odds ratios of 0.29 and 0.74 respectively). The reasons for these geographical differences are unclear, but might include different approaches to recruitment by staff. However, this does not explain the difference in participation between UCH and the Brompton because the same research nurses were used for both centres.

Appendix Table S4. End of trial status, including the exit chest radiography

	**Control group**<br>**N=783**	**Screened group**<br>**N=785**
Lung cancer	36 (5%)	42 (5%)
Other cancers	51 (7%)	47 (6%)
Deaths	96 (12%)	70 (9%)
Lung cancer	21	16
Other cancer	17	14
All other causes	48	38
Unknown cause	10	2
**Smoking status:**		
Current smoker that continued	242 (31%)	220 (28%)
Current smoker that reduced	46 (6%)	55 (7%)
Current smoker that stopped	51 (7%)	59 (8%)
Ex-smoker no change	284 (36%)	277 (35%)
Ex-smoker re-started	7 (1%)	3 (0%)
unknown/missing	*153 (20%)*	*171 (22%)*
**Exit chest radiography:**		
At end of 5 years	451	393
Before 5 years (among withdrawals)	35	37

Because the hospital respiratory units recruited trial participants and so had an interest in the study through their lead clinical investigator, it is possible they were more proactive with managing these particular participants. However, the percentage of lung cancers found among those recruited from general practice/family physicians (4.6%, 57/1241) did not significantly differ from the hospitals (6.4%, 21/327), p=0.22.

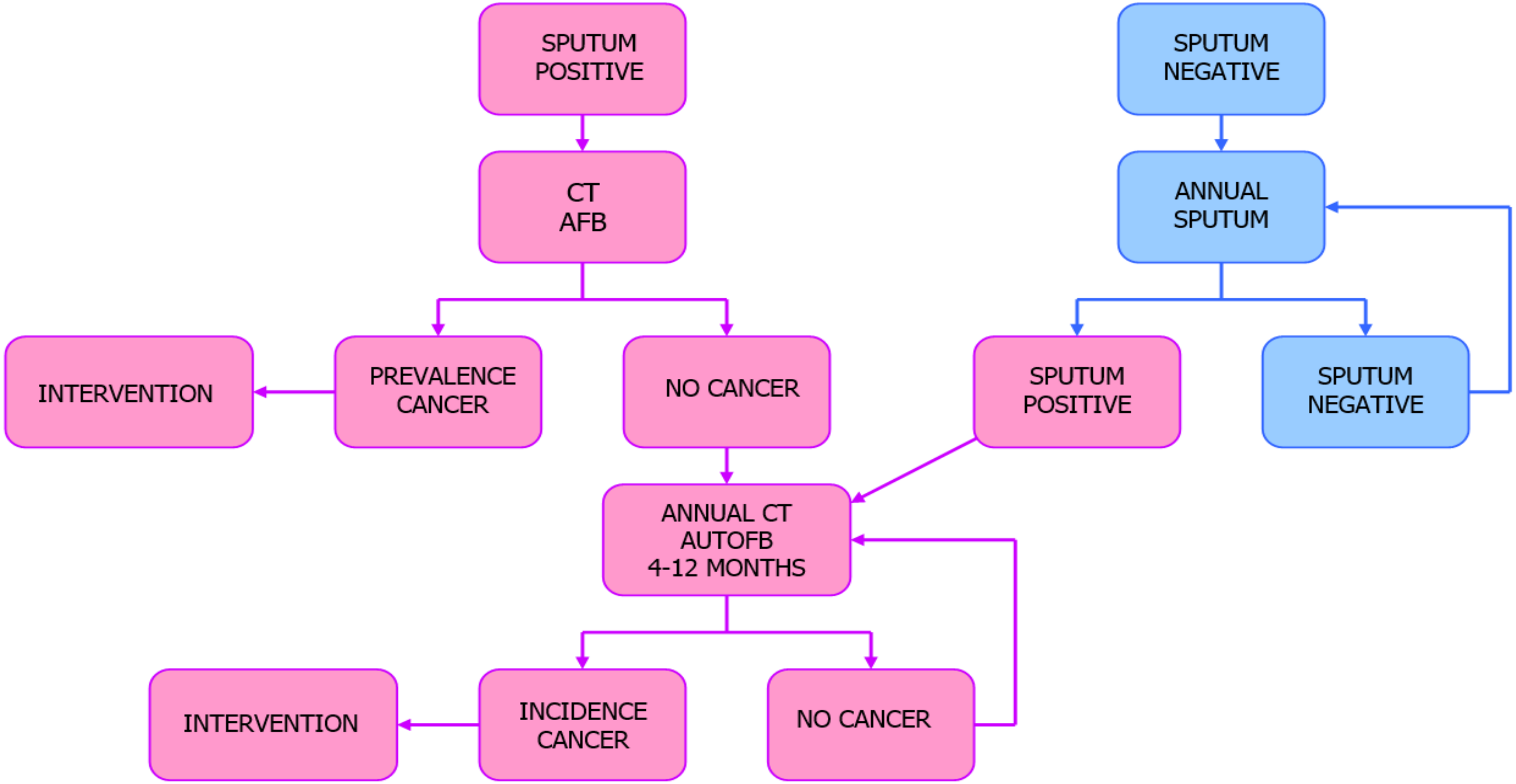

Appendix Figure S1. Flow diagram for trial participants in the screened arm. CT (low dose spiral CT scan), AFB (auto-florescence bronchoscopy)

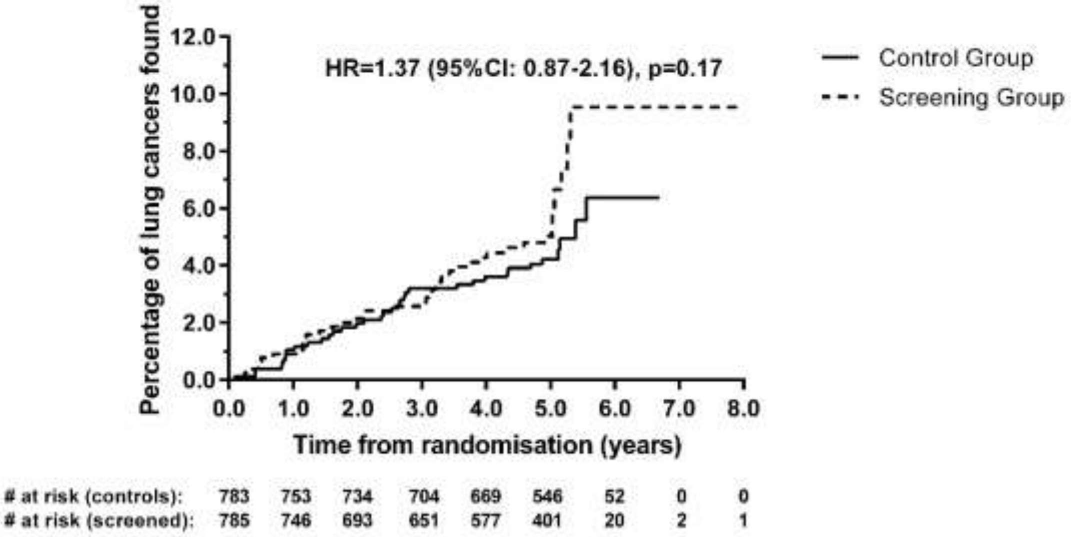

Appendix Figure S2. Kaplan-Meier plot for the incidence of lung cancer.
The apparent increase in the risk of lung cancer diagnosis after 5 years is mainly due to the size of the steps in the Kaplan-Meier plot being exaggerated because there are relatively few individuals followed up for this long, with very few events. The trial protocol specified 5 years follow up, a few patients appeared to have longer than this mainly because of flexibility given to the date of their exit scans.

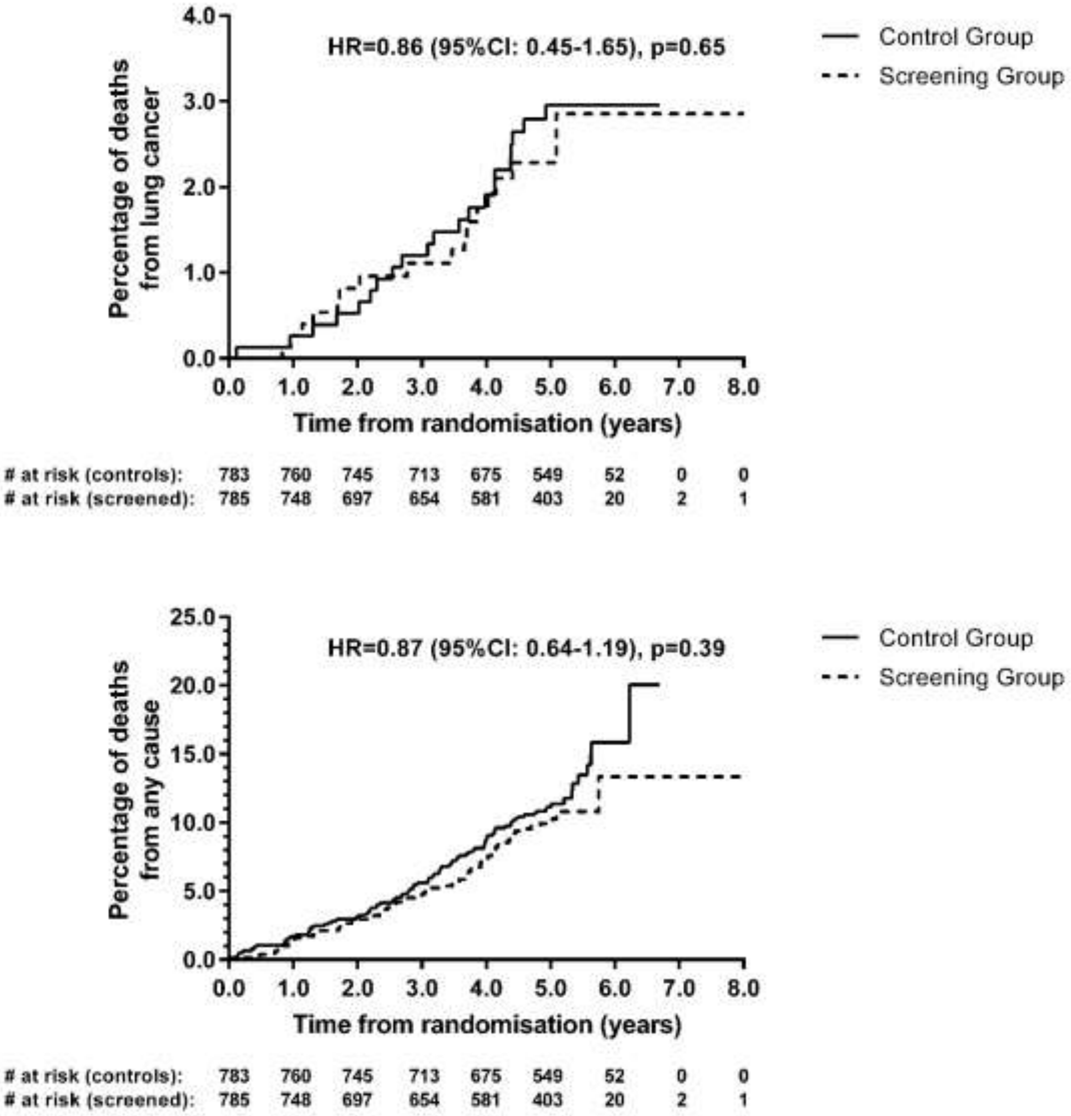

Appendix Figure S3. Kaplan-Meier plots for deaths due to lung cancer (upper), and all cause mortality (lower)
