# Supplementary material for: Sequential screening for lung cancer in a high-risk group: randomised controlled trial: LungSEARCH: a randomised controlled trial of Surveillance using sputum and imaging for the EARly detection of lung Cancer in a High-risk group
Source: Eur Respir J. 2019 Oct 17;54(4):1900581. doi: 10.1183/13993003.00581-2019 (PMC6796151; doi:10.1183/13993003.00581-2019)
Supplement: Supplementary file 2 [file ERJ-00581-2019.Shareable.pdf]

ORIGINAL ARTICLE
LUNG CANCER

SHAREABLE PDF

Sequential screening for lung cancer in a high-risk group: randomised controlled trial

LungSEARCH: a randomised controlled trial of Surveillance using sputum and imaging for the EARly detection of lung Cancer in a High-risk group

Stephen G. Spiro[1,22], Pallav L. Shah[2], Robert C. Rintoul[3], Jeremy George[4], Samuel Janes[4], Matthew Callister[5], Marco Novelli[6], Penny Shaw[7], Gabrijela Kocjan[6], Chris Griffiths[8], Mary Falzon[6], Richard Booton[9], Nicholas Magee[10], Michael Peake[11,12], Paul Dhillon[13], Kishore Sridharan[14], Andrew G. Nicholson[15], Simon Padley[16], Magali N. Taylor[7], Asia Ahmed[7], Jack Allen[17], Yenting Ngai[17], Nyasha Chinyanganya[17], Victoria Ashford-Turner[18], Sarah Lewis[19], Dahmane Oukrif[20], Pamela Rabbitts[21], Nicholas Counsell[17] and Allan Hackshaw[17,22]

Affiliations: [1]Dept of Respiratory Medicine, University College Hospital, London, UK. [2]Dept of Respiratory Medicine, Royal Brompton Hospital, Chelsea and Westminster Hospital and Imperial College London, London, UK. [3]Dept of Oncology, Royal Papworth Hospital and University of Cambridge, Cambridge, UK. [4]UCL Respiratory, Dept of Medicine, University College London, London, UK. [5]Dept of Respiratory Medicine, Leeds Teaching Hospitals NHS Trust, Leeds, UK. [6]Cellular Pathology, University College Hospital, London, UK. [7]Radiology (Imaging), University College Hospital, London, UK. [8]Institute of Population Health Sciences, Barts and the London School of Medicine and Dentistry, Queen Mary University of London, London, UK. [9]Lung Cancer and Thoracic Surgery Directorate, Manchester University NHS Trust and University of Manchester, Manchester, UK. [10]Respiratory Medicine, Belfast City Hospital, Belfast, UK. [11]Dept of Immunity, Infection and Inflammation, University of Leicester, Leicester, UK. [12]Centre for Cancer Outcomes, University College London Hospitals NHS Foundation Trust, London, UK. [13]Respiratory Medicine, University Hospitals Coventry and Warwickshire, Coventry, UK. [14]Dept of Thoracic Medicine, Sunderland Royal Hospital, Sunderland, UK. [15]Dept of Histopathology, Royal Brompton Hospital and Harefield NHS Foundation Trust and National Heart and Lung Institute, London, UK. [16]Radiology, Royal Brompton Hospital and National Heart and Lung Institute, Imperial College London, London, UK. [17]Cancer Research UK and UCL Cancer Trials Centre, London, UK. [18]Cardio-Respiratory Medicine, Leeds Teaching Hospitals NHS Trust, Leeds, UK. [19]Research and Development, Royal Papworth Hospital, Cambridge, UK. [20]Dept of Pathology, University College Hospital, London, UK. [21]Leeds Institute of Cancer and Pathology (LICAP), University of Leeds, Leeds, UK. [22]These authors are joint lead authors.

Correspondence: Allan Hackshaw, Cancer Research UK and UCL Cancer Trials Centre, 90 Tottenham Court Road, London, W1T 4TJ, UK. E-mail: a.hackshaw@ucl.ac.uk

@ERSpublications
While low-dose CT is now preferred for lung cancer screening, our randomised trial of smokers with COPD showed that a proposed sequential policy using sputum testing to select who receives low-dose CT and autofluorescence bronchoscopy was ineffective http://bit.ly/2JZujnx

Cite this article as: Spiro SG, Shah PL, Rintoul RC, *et al*. Sequential screening for lung cancer in a high-risk group: randomised controlled trial. *Eur Respir J* 2019; 54: 1900581 [https://doi.org/10.1183/13993003.00581-2019].

This single-page version can be shared freely online.

ABSTRACT
Background: Low-dose computed tomography (LDCT) screening detects early-stage lung cancer and reduces mortality. We proposed a sequential approach targeted to a high-risk group as a potentially efficient screening strategy.
Methods: LungSEARCH was a national multicentre randomised trial. Current/ex-smokers with mild/

Copyright ©ERS 2019. This article is open access and distributed under the terms of the Creative Commons Attribution Licence 4.0.

moderate chronic obstructive pulmonary disease (COPD) were allocated (1:1) to have 5 years surveillance or not. Screened participants provided annual sputum samples for cytology and cytometry, and if abnormal were offered annual LDCT and autofluorescence bronchoscopy (AFB). Those with normal sputum provided annual samples. The primary end-point was the percentage of lung cancers diagnosed at stage I/II (nonsmall cell) or limited disease (small cell).

Results: 1568 participants were randomised during 2007–2011 from 10 UK centres. 85.2% of those screened provided an adequate baseline sputum sample. There were 42 lung cancers among 785 screened individuals and 36 lung cancers among 783 controls. 54.8% (23 out of 42) of screened individuals *versus* 45.2% (14 out of 31) of controls with known staging were diagnosed with early-stage disease (one-sided p=0.24). Relative risk was 1.21 (95% CI 0.75–1.95) or 0.82 (95% CI 0.52–1.31) for early-stage or advanced cancers, respectively. Overall sensitivity for sputum (in those randomised to surveillance) was low (40.5%) with a cumulative false-positive rate (FPR) of 32.8%. 55% of cancers had normal sputum results throughout. Among sputum-positive individuals who had AFB, sensitivity was 45.5% and cumulative FPR was 39.5%; the corresponding measures for those who had LDCT were 100% and 16.1%, respectively.

Conclusions: Our sequential strategy, using sputum cytology/cytometry to select high-risk individuals for AFB and LDCT, did not lead to a clear stage shift and did not improve the efficiency of lung cancer screening.
